# The Consistency and Quality of ChatGPT Responses Compared to Clinical Guidelines for Ovarian Cancer: A Delphi Approach

**DOI:** 10.3390/curroncol31050212

**Published:** 2024-05-14

**Authors:** Dario Piazza, Federica Martorana, Annabella Curaba, Daniela Sambataro, Maria Rosaria Valerio, Alberto Firenze, Basilio Pecorino, Paolo Scollo, Vito Chiantera, Giuseppe Scibilia, Paolo Vigneri, Vittorio Gebbia, Giuseppa Scandurra

**Affiliations:** 1 Medical Oncology Unit, Casa di Cura Torina, 90145 Palermo, Italy; dariopiazza@casadicuratorina.it (D.P.); annabella.curaba@gmail.com (A.C.); 2 Department of Clinical and Experimental Medicine, University of Catania, 95124 Catania, Italy; fede.marto.fm@gmail.com; 3 Medical Oncology Unit, Ospedale Umberto I, 94100 Enna, Italy; danielasambataro1@gmail.com; 4 Medical Oncology Unit, Policlinico P. Giaccone, University of Palermo, 90133 Palermo, Italy; valerionc17@gmail.com; 5 Occupational Health Section, Department of Health Promotion, Mother and Child Care, Internal Medicine and Medical Specialties, University of Palermo, 90133 Palermo, Italy; alberto.firenze@unipa.it; 6 Gynecology Unit, Ospedale Cannizzaro, 95126 Catania, Italy; basilio.pecorino@unikore.it (B.P.); paolo.scollo@unikore.it (P.S.); 7 Gynecology, Faculty of Medicine and Surgery, University of Enna Kore, 94100 Enna, Italy; 8 Gynecology, University of Palermo, 90133 Palermo, Italy; vito.chiantera@gmail.com; 9 Gynecology Unit, Ospedale Paternò Arezzo, 97100 Ragusa, Italy; g.scibilia@libero.it; 10 Medical Oncology, University of Catania, 95124 Catania, Italy; vigneripaolo@gmail.com; 11 Medical Oncology, Istituto Clinico Humanitas, 95045 Catania, Italy; 12 Medical Oncology, Faculty of Medicine and Surgery, University of Enna Kore, 94100 Enna, Italy; 13 Medical Oncology Unit, Ospedale Cannizzaro, 95126 Catania, Italy; giusy.scandurra@gmail.com

**Keywords:** artificial intelligence, ChatGPT, ovarian carcinoma, guidelines

## Abstract

Introduction: In recent years, generative Artificial Intelligence models, such as ChatGPT, have increasingly been utilized in healthcare. Despite acknowledging the high potential of AI models in terms of quick access to sources and formulating responses to a clinical question, the results obtained using these models still require validation through comparison with established clinical guidelines. This study compares the responses of the AI model to eight clinical questions with the Italian Association of Medical Oncology (AIOM) guidelines for ovarian cancer. Materials and Methods: The authors used the Delphi method to evaluate responses from ChatGPT and the AIOM guidelines. An expert panel of healthcare professionals assessed responses based on clarity, consistency, comprehensiveness, usability, and quality using a five-point Likert scale. The GRADE methodology assessed the evidence quality and the recommendations’ strength. Results: A survey involving 14 physicians revealed that the AIOM guidelines consistently scored higher averages compared to the AI models, with a statistically significant difference. Post hoc tests showed that AIOM guidelines significantly differed from all AI models, with no significant difference among the AI models. Conclusions: While AI models can provide rapid responses, they must match established clinical guidelines regarding clarity, consistency, comprehensiveness, usability, and quality. These findings underscore the importance of relying on expert-developed guidelines in clinical decision-making and highlight potential areas for AI model improvement.

## 1. Introduction

Ovarian cancer (OC) is a significant worldwide health concern, with high mortality rates and few therapeutic options. OC is the fifth most common malignancy, ranking fourth among cancer-related deaths in women in the USA, and is the leading cause of gynecologic cancer-related death in the Western world [1]. In Italy, OC ranks tenth among all female cancers (3%), with approximately 5200 new diagnoses in 2020, 3200 deaths in 2021, and a 5-year net survival rate of 43% from the time of diagnosis [2]. 

International and national guidelines have been developed to endow evidence-based recommendations for the diagnosis, treatment, and follow-up of OC cancer patients. The National Comprehensive Cancer Network (NCCN) and the European Society of Medical Oncology (ESMO) guidelines elaborate and constantly update evidence-based recommendations for managing OC [3,4]. The Italian Association of Medical Oncology (AIOM) has also developed guidelines to provide evidence-based recommendations for OC patients’ diagnosis, treatment, and follow-up [5].

ChatGPT (Generative Pre-trained Transformer) is a natural language artificial intelligence model developed by OpenAI based on the transformer architecture [6,7]. The first version, i.e., GPT-3.5, was a potent model capable of understanding context and generating highly accurate responses. However, with the introduction of GPT-4, the model’s capabilities have been significantly enhanced. GPT-4 has substantially increased the model size and the number of parameters, making it more accurate in understanding context and capable of generating creative and coherent responses [8]. Moreover, thanks to improved training and the algorithm, GPT-4 has become more efficient in handling user queries, providing better natural language interpretation, even in complex situations. Despite being based on the same architecture as its predecessor, GPT-4 represents a significant step forward in artificial intelligence and natural language processing [9]. Given its capabilities, ChatGTP may have significant applications in several medical fields, including oncology. It could provide immediate responses to frequently asked questions, freeing time for medical professionals to focus on more complex tasks [10]. In oncology, GPT-4 could interpret patient data, helping doctors understand symptom patterns and trends or treatment responses [10,11].

Furthermore, GPT-4 could assist health professionals in providing personalized reports on medical status, treatment options, and potential side effects to patients [10,11,12,13]. This tool could enhance patient understanding and decision-making, promoting patient-centered care [14]. However, using AI in patient care should always be coupled with appropriate ethical considerations, including regarding privacy, accuracy, and transparency [14,15]. Additionally, using such tools in the medical field raises doubts and concerns about the accuracy and reliability of the information provided. 

We conducted a study to investigate the consistency and quality of responses generated by OpenAI’s language model—ChatGPT—to clinical queries concerning OC, comparing the results to the Italian guidelines. The evaluations focused on the clarity of recommendations, the relevance of the evidence presented, the comprehensiveness of the information, and applicability in clinical practice. The study provides comparisons of AI-generated clinical advice with established oncology guidelines, thereby assessing the utility and validity of AI in facilitating healthcare.

## 2. Materials and Methods

### 2.1. Study Design

**Figure 1:**
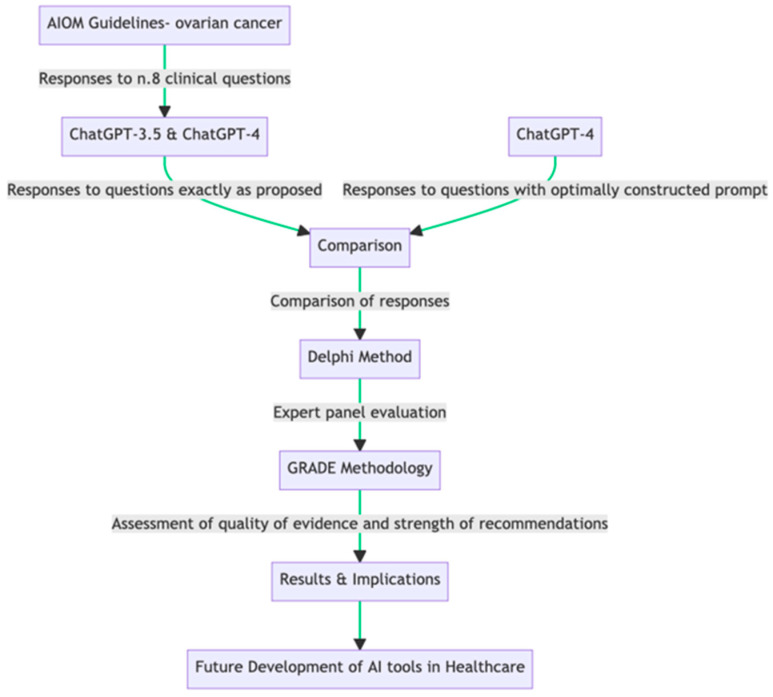
Flowchart of the study design.

In this study, we employed a rigorous approach to evaluate the consistency and quality of responses generated by OpenAI’s ChatGPT to clinical queries related to OC treatment, compared to the guidelines published by the Italian Association of Medical Oncology (AIOM). The latter guidelines offered responses to eight clinical questions, and these identical queries were posed to two versions of the ChatGPT model, 3.5 and 4 (Table 1a). An additional set of queries was presented to ChatGPT model 4, with an optimally constructed prompt designed to elicit structured responses. Three rounds of questioning were conducted for each model and query type, replicating the real-world variability in question presentation (Table 1b). The responses from these models were then compared with those outlined in the AIOM guidelines. These comparisons were carried out quantitatively by comparing the direct similarities and differences in the given advice and qualitatively by assessing the clarity, consistency, comprehensiveness, and usability of the information provided by the AI models (Figure 1). To perform this evaluation, we applied the Delphi method, which involves a panel of experts participating in iterative rounds of evaluation until a consensus is reached [16]. Our expert panel comprised diverse healthcare professionals and researchers, including oncologists, gynecologists, pathologists, radiologists, and evidence-based medicine experts. The experts assessed the AI responses using a 5-point Likert scale based on predefined criteria.

Furthermore, we used the GRADE methodology to assess the quality of evidence and the strength of the recommendations given by the AI models. GRADE is a systematic approach that helps to assess the quality of evidence in studies and the strength of health care recommendations [17]. This methodology was used to assess both the responses given by ChatGPT and the responses provided by the AIOM guidelines. Due to the nature of the study, approval by the Ethics Committee and the Informed Consent Statement were waived according to Italian law.

**Table 1:** (**a**) The AIOM ovary guidelines’ eight clinical questions. (**b**) The format of how questions are proposed concerning the model used.

(a)	
n.#	Clinical Question
1	In patients with advanced epithelial carcinoma of the ovary undergoing complete macroscopic resection and with negative lymph nodes on imaging and intraoperative evaluation (P), is systematic lymphadenectomy (I) recommended over non lymphadenectomy (C) in terms of overall survival, PFS, quality of life, and complications (O)?
2	In patients with advanced epithelial carcinoma of the ovary, stage IIIC-IV (P) is primary surgery (I) recommended over neoadjuvant chemotherapy followed by interval surgery (C) in terms of overall survival, PFS, quality of life, and complications (O)?
3	In patients with platinum-sensitive recurrence of epithelial carcinoma of the ovary (P), is cytoreductive surgery followed by chemotherapy (I) recommended over chemotherapy alone (C) in terms of overall survival, PFS, and complications (O)?
4	In patients with FIGO stage IIIB-IV ovarian cancer (P), is bevacizumab administration in combination and maintenance at the end of first-line chemotherapy (I) recommended compared with chemotherapy alone (C) in terms of overall survival (OS), progression-free survival (PFS), and complications (O)?
5	In patients with low-grade FIGO stage II-IV serous ovarian cancer (P), is maintenance hormone therapy recommended at the end of first-line platinum-based chemotherapy (I) compared with no maintenance (C) in terms of overall survival (OS), progression-free survival (PFS), and complications (O)?
6	In BRCA-mutated patients with high-grade FIGO stage III-IV serous ovarian and endometrioid cancer (P), is maintenance therapy with Olaparib at the end of first-line platinum-based chemotherapy (I) recommendable compared with non maintenance (C) in terms of PFS, time to next chemotherapy, time to second subsequent progression (PFS2), quality of life, overall survival, and tolerability (O)?
7	In patients with high-risk FIGO stage III-IV (P) serous and endometrioid ovarian cancer, is maintenance therapy with Niraparib at the end of first-line platinum-based chemotherapy (I) recommendable compared with non maintenance (C) in terms of PFS, time to next chemotherapy, time to second subsequent progression (PFS2), quality of life, overall survival, and tolerability?
8	In patients with stage I (P) immature teratoma, is adjuvant treatment (I) recommended over no treatment (C) in terms of overall survival (OS), disease-free survival (DFS), and tolerability (O)?
**(b)**	
**Model**	**Prompt**
ChatGPT-3.5	[Clinical Question #] * (as proposed from source document)
ChatGPT-4	[Clinical Question #] * (as proposed from source document)
ChatGPT-4	Act as an Italian multidisciplinary oncology group. We ask a question using the PICO method. Reply extensively based on national and international guidelines and current evidence, indicate the limitations of the evidence, and indicate the ratio of benefits to harms. Also, provide answers with a formal GRADE approach indicating the overall quality of evidence and strength of recommendation. ^§^ [Clinical Question #] *

* Questions asked in the same language as in the source document. ^§^ Prompt structured and proposed in the same language as in the source document.

### 2.2. Statistics

One-way ANOVA test was used to compare the mean scores of results. A Tukey post hoc test was carried out to identify which groups significantly differed.

## 3. Results

The survey was conducted among fourteen physicians, seven oncologists, and seven gynecologists, who thoroughly evaluated the responses to the eight clinical questions in five main domains: clarity, consistency, comprehensiveness, usability, and quality. Table 2 shows the evaluation questions grouped by domains and their average values. The AIOM guidelines consistently scored higher averages compared to the artificial intelligence models.

We performed a one-way ANOVA test to compare the mean scores across the AI models and the guidelines. The test showed a significant difference between groups (F = 21.66, *p* < 0.00001), suggesting that at least one of the groups differed significantly from the others. Following the ANOVA results, a Tukey post hoc test was carried out to identify which groups significantly differed. The test showed that the AIOM guidelines significantly differed from all other groups (ChatGPT-3.5, ChatGPT-4, and ChatGPT-4 with a prompt), with an adjusted *p*-value for multiple comparisons below 0.05. Among the artificial intelligence models, there was no significant difference between ChatGPT-3.5 and ChatGPT-4 or between ChatGPT-4 and ChatGPT-4 with a prompt (Table 3).

**Table 2:** Survey assessment questions and average results.

Domains	Questions	Mean	CI (±95%)
clarity	How do you think the guideline expresses its recommendations?	4.28	0.14
	How does the ChatGPT-3.5 model’s response to the clinical question express its recommendations?	1.23	0.12
	How does the ChatGPT-4 model’s response to the clinical question express its recommendations?	2.23	0.21
	How does the prompted ChatGPT-4 model’s response to the clinical question express its recommendations?	3.31	0.21
relevance	How relevant is the evidence in the guideline for the recommendations?	4.35	0.15
	How relevant is the evidence presented in the ChatGPT-3.5 model’s response to the clinical question for the recommendations made?	1.36	0.09
	How relevant is the evidence presented in the ChatGPT-4 model’s response to the clinical question for the recommendations made?	2.25	0.24
	How relevant is the evidence presented in the prompted ChatGPT-4 model’s response to the clinical question for the recommendations made?	3.15	0.24
comprehensiveness	How comprehensive are the guidelines in addressing the topic?	4.53	0.13
	How comprehensive is the ChatGPT-3.5 model’s response to the clinical question in addressing the topic?	1.11	0.06
	How comprehensively does the ChatGPT-4 model’s response to the clinical question is in addressing the topic?	2.13	0.22
	How comprehensive is the prompted ChatGPT-4 model’s response to the clinical question in addressing the topic?	2.95	0.23
applicability	How applicable is the guide to clinical practice?	4.28	0.14
	How applicable is the ChatGPT-3.5 model’s response to the clinical question to clinical practice?	1.23	0.12
	How applicable is the ChatGPT-4 model’s response to the clinical question to clinical practice?	2.26	0.23
	How applicable is the prompted ChatGPT-4 model’s response to the clinical question to clinical practice?	2.82	0.27
quality	According to the GRADE approach, how would you rate the strength of the recommendations and the quality of the evidence presented in the guideline?According to the GRADE approach	2.3	0.16
	How would you rate the recommendations’ strength and the evidence’s quality presented in the ChatGPT-3.5 model’s response?According to the GRADE approach	1.88	0.12
	How would you rate the recommendations’ strength and the evidence’s quality presented in the ChatGPT-4 model’s response?According to the GRADE approach	2.49	0.26
	How would you rate the recommendations’ strength and the evidence’s quality presented in the prompted ChatGPT-4 model’s response?	2.38	0.26

**Table 3:** Tukey post hoc test results.

Domain 1	Domain 2	Mean Difference	Adjusted *p*-Value	Lower Bound	Upper Bound	Reject Null Hypothesis
ChatGPT-3.5	ChatGPT-4	0.91	0.0618	−0.037	1.857	False
ChatGPT-3.5	Guidelines	2.586	0.001	1.639	3.533	True
ChatGPT-3.5	Prompted ChatGPT-4	1.56	0.0012	0.613	2.507	True
ChatGPT-4	Guidelines	1.676	0.001	0.729	2.623	True
ChatGPT-4	Prompted ChatGPT-4	0.65	0.242	−0.297	1.597	False
Guidelines	Prompted ChatGPT-4	−1.026	0.0314	−1.973	−0.079	True

## 4. Discussion

Recently, there has been increasing interest in incorporating AI into healthcare education, research, and clinical practice. One AI-based tool that has gained traction is ChatGPT, a large language model that can provide professional support to patients, medical professionals, researchers, and educators. Several studies have investigated the potential applications and limitations of ChatGPT in medicine. Yeo et al. assessed the performance of ChatGPT in answering queries concerning cirrhosis and hepatocellular carcinoma (HCC). Their study showed that ChatGPT regurgitated extensive knowledge of cirrhosis and HCC, but only small proportions were labeled comprehensive [18]. Similarly, another study evaluated the feasibility of ChatGPT in healthcare and analyzed several clinical and research scenarios [19]. Results indicated that while AI-based language models like ChatGPT have impressive capabilities, they may perform poorly in real-world settings, especially medicine, where high-level and complex thinking is necessary. 

Recently, the scientific community has raised ethical concerns about using ChatGPT to write scientific articles and other scientific output. A recent systematic review was conducted to investigate the utility of ChatGPT in healthcare [20]. The researchers retrieved 60 records that examined ChatGPT in the context of healthcare education, research, or practice. Their findings highlighted the benefits of ChatGPT, which included improved scientific writing, enhanced research equity and versatility, utility in healthcare research, and time-saving, allowing greater focus on experimental design and downstream analysis. However, the authors also emphasized the need to address valid concerns associated with ChatGPT in healthcare, such as data protection and the potential negative impacts on physician–patient relationships. Kim et al. discussed the current acceptability of ChatGPT and large language model (LLM) chatbots in academic medicine and proposed guidelines for their utilization [21]. They identified the potential benefits of using ChatGPT and LLM chatbots, such as increased access to healthcare information and support. They also highlighted the challenges that need to be addressed, such as data privacy and the impact on medical professionalism. 

The use of ChatGPT in oncology care has gained considerable attention in recent months. In an observational study, ChatGPT was evaluated for its ability to identify guideline-based treatments for advanced solid tumors [22]. The study demonstrated that ChatGPT can elaborate upon appropriate therapeutic choices for new diagnoses of advanced solid malignancies through standardized prompts. The valid therapy quotient (VTQ) was introduced as a ratio of medications listed by ChatGPT to those suggested in the NCCN guidelines, revealing that ChatGPT correctly identified guideline-based treatments in about 70% of cases. In a recent editorial, Kothari revealed that ChatGPT attracted many active users quickly due to its extraordinary ability to understand and generate human-like language [23]. In addition, ChatGPT has generated various types of content, including scholarly work, exam questions, and discharge summaries. Hamilton et al. evaluated the clinical relevance and accuracy of ChatGPT-generated next-generation sequencing (NGS) reports with first-line treatment recommendations for NSCLC patients with targetable driver oncogenes [24]. The study concluded that ChatGPT-generated reports were contextually accurate and clinically relevant. 

Although the potential benefits of ChatGPT in healthcare are significant, researchers continue to investigate the technology’s integration and effectiveness across diverse fields. Cheng et al. discussed how the integration of ChatGPT can enable a new era of surgical oncology [25], while Ebrahimi et al. evaluated whether a natural language processing tool like ChatGPT would be trustworthy for radiation oncology use [26]. A study by Haemmerli et al. evaluated the ChatGPT recommendations for glioma management with a panel of CNS tumor experts [27]. The CNS tumor board experts assessed ChatGPT and found that while it performed poorly in diagnosing glioma types, it performed well in recommending adjuvant treatments. Despite its inability to match the accuracy of expert judgments, ChatGPT shows promise as an additional tool when used in conjunction with a human in the loop. Huang et al. assessed the potential of ChatGPT-4 for AI-assisted medical education and decision-making in radiation oncology [28]. While noting ChatGPT-4’s limits in some areas, the study showed the technology’s potential for clinical decision support and medical education of the public and cancer patients. However, because of the possibility of generating false information, confirming the authenticity of the content produced by models like ChatGPT is crucial.

This paper is the first report comparing ChatGPT outputs to clinical guideline recommendations in oncology. Our study assessed the responses to eight clinical questions provided by the AIOM guidelines on ovarian cancer and three generative artificial intelligence models, ChatGPT-3.5, ChatGPT-4, and ChatGPT-4, with a structured prompt. A multidisciplinary team evaluated the responses across five main domains, clarity, consistency, comprehensiveness, usability, and quality, using a five-point Likert scale. The resulting scores across the domains indicate that the AIOM guidelines consistently achieved higher mean scores than the generative artificial intelligence models. This report suggests that the physicians surveyed found the responses provided by the AIOM guidelines to be more precise, relevant, comprehensive, applicable, and of higher quality than those provided by the AI models. Medical experts developed medical-scientific guidelines based on extensive research and consensus among the medical community. At the same time, AI models, despite their advanced capabilities, may still need more subtlety and depth of understanding inherent in human expertise. The results of the one-way ANOVA test further support this observation, revealing a significant difference between the groups. These data suggest a statistically significant variation in the mean scores between at least one group pair, reinforcing the conclusion that the AIOM guidelines were evaluated more favorably. The Tukey post hoc test, conducted to identify which specific groups differed significantly, indicated that the AIOM guidelines significantly differed from all other groups. Interestingly, there were no significant differences among the artificial intelligence models, suggesting that adding a structured prompt in ChatGPT-4 did not significantly enhance its performance in this context.

## 5. Limitations

While ChatGPT and other AI-based tools hold promise in healthcare education, research, and practice, it is essential to recognize and address their limitations and potential ethical concerns. Correct information and users’ education on the appropriate use and potential pitfalls of AI-based language models are crucial to ensure that they are used to optimize their benefits while minimizing any potential harm.

This study may have limitations due to the small sample size of the physicians surveyed, which may impact the generalizability of the results. In addition, the fact that the study is based solely on Italian national guidelines may limit the scope of recommendations and overlook potentially valuable guidance from other international best practices or specialized institutions. Finally, the use of ChatGPT exclusively as an artificial intelligence tool may raise concerns about the completeness and accuracy of the responses, as it lacks comparison with other currently available tools.

## 6. Conclusions

The future of new generative artificial intelligence tools in the medical field is promising, potentially improving the quality and consistency of medical information provided to patients. However, ensuring that the information provided is accurate and reliable is essential, nevertheless further research is needed to evaluate their effectiveness and address concerns about their accuracy and reliability. In conclusion, while AI models such as ChatGPT can provide rapid responses to clinical questions, our study suggests they must match up to established clinical guidelines regarding clarity, relevance, comprehensiveness, applicability, and quality, as oncologists and gynecologists perceive them. These observations underscore the importance of relying on expert-developed guidelines in clinical decision-making while highlighting potential areas for improvement in AI models for clinical use. Tracking how these comparisons may change over time will be interesting as AI evolves.

## Data Availability

The data presented in this study are available from the corresponding author upon request.
